# Immune functions during chronic viral infections

**DOI:** 10.1186/2047-783X-19-S1-S25

**Published:** 2014-06-19

**Authors:** Philipp Lang, Dieter Häussinger, Karl S Lang

**Affiliations:** 1 Clinic of Gastroenterology, Hepatology, and Infectious Diseases, Heinrich Heine University, 40225 Düsseldorf, Germany; 2 Institute for Immunology, University of Duisburg-Essen, 45147 Essen, Germany

## 

Chronic viral infections display a major health burden worldwide. Immune control of chronic viral infections requires a balanced interplay between immune activation promoting virus clearance and deactivation of anti-viral immunity to prevent tissue damage. Our research is focused on identifying mechanisms regulating the balanced immune control of viral infections.

Innate type I interferon production can limit viral replication in infected cells thereby contributing to elimination of viruses. Our data demonstrate that IRF7 is a key transcription factor for systemic type I interferon production. Furthermore, macrophages play a key role in limiting a viral infection after exposure to type I interferon and prevent the infection from becoming chronic [1]. Type I Interferon can activate also other immune cells such as natural killer (NK) cells. However, the role of NK cells during chronic viral infection remains insufficiently understood. Also during infection with lymphocytic choriomeningitis virus, NK cell cytotoxicity can be detected. Furthermore, while anti-viral CD8^+^ T cells can lose their function during establishment of a chronic viral infection, a mechanism termed T cell exhaustion, NK cell depletion restored T cell function in virus infected animals and prevented chronic viral infection. These effects were mediated by the effector protein perforin [2].

Furthermore, during viral induced hepatitis using the lymphocytic choriomeningitis virus, platelet infiltrates could be observed in snap frozen liver sections. Platelets readily release serotonin after activation. Consistently, liver blood flow was highly reduced during viral infection in control animals, but not in serotonin deficient mice. Consequently, CD8^+^ T cell immunity in liver tissue of serotonin deficient mice was enhanced and able to eliminate the viral infection faster than corresponding control animals. This resulted in reduced virus induced T cell mediated liver cell damage during viral infection [3].

Moreover, during virus induced liver infection, macrophages and neutrophils exhibited production of reactive oxygen species (ROS), which was dependent on the subunit of the NAPDH oxidase p47phox. Limited ROS production in p47phox deficient mice resulted in enhanced T cell survival and elevated T cell function in liver tissue. Consequently, liver cell damage was highly reduced in absence of the NADPH oxidase regulating subunit p47phox [4].

Taken together these data indicates novel mechanisms during establishment of a chronic viral infection as well as viral induced hepatitis.
